# Early menarche is associated with disordered eating—results from a National Youth Survey

**DOI:** 10.1038/s41390-025-03852-1

**Published:** 2025-01-16

**Authors:** Sharon Iron-Segev, Chen Namimi-Halevi, Chen Dor, Rita Dichtiar, Aliza H. Stark, Lital Keinan-Boker, Tali Sinai

**Affiliations:** 1 Israel Center for Disease Control, Israel Ministry of Health, Ramat Gan, Israel; 2 School of Nutritional Sciences, The Robert H. Smith Faculty of Agriculture, Food and Environment, The Hebrew University of Jerusalem, Rehovot, Israel; 3 The Faculty of Social Welfare and Health Sciences, University of Haifa, Haifa, Israel

## Abstract

**Background:**

Disordered eating (DE) is highly prevalent among adolescents, though its definition varies. The association between DE and early pubertal maturation (EPM) remains underexplored in Israel, and has not been sufficiently examined using the widely-used SCOFF questionnaire. This study examines these associations in adolescents.

**Methods:**

Participants (*n* = 2415 girls, 2095 boys; ages 12–18 years) in a nationally-representative, cross-sectional Youth Health and Nutrition Survey (2015–2016) completed self-administered questionnaires, including the SCOFF questionnaire, and underwent anthropometric measurements. EPM was determined by menarcheal age <11.5 years in girls, and facial hair appearance <12.5 years in boys. Respondents affirming ≥2 SCOFF items were classified as DE cases. Multivariable logistic regression analyses examined the associations between EPM and DE.

**Results:**

Among the participants, 12.7% of the girls and 20.4% of the boys met EPM criteria; 55.5% and 33.7%, respectively, were categorized as having DE. Following adjustment for age, socioeconomic status, ethnic background, and weight status, EPM was significantly associated with DE in girls (OR 1.47, 95%CI: 1.12–1.93) and with 3/5 SCOFF items. No such association was found in boys (OR 1.00, 95%CI: 0.77–1.28).

**Conclusion:**

EPM in girls was associated with DE. Identifying high risk groups for DE in adolescents is crucial for early intervention and prevention.

**Impact:**

- This study included a large, nationally representative sample of Israeli adolescents and utilized the SCOFF questionnaire, a widely used screening measure for disordered eating (DE).
- Following comprehensive analyses, a significant association between early pubertal maturation (EPM), defined as early menarche, and DE was documented in Israeli adolescent girls.
- Identifying girls with EPM and screening for disordered eating will allow for early interventions, potentially improving physical and mental health, and preventing progression to eating disorders.

## Introduction

Disordered eating (DE) refers to abnormal eating or weight-control behaviors that fall below the threshold for clinically diagnosed eating disorders. These behaviors, including dieting, fasting, binge eating, meal skipping, and self-induced vomiting, are often harmful and ineffective attempts to control weight or achieve a lean appearance.^1^ DE is associated with both physical and psychological consequences, and a reduced quality of life. Children and adolescents with DE are at higher risk for presenting disturbed eating in young adulthood.^2,3^

Epidemiological studies indicate that DE is far more common than clinical eating disorders.^1^ Previous reports on the prevalence of DE symptoms among Israeli youth of both sexes were in the range of 20–30%.^4,5^ DE affects individuals across various demographics, including different ages, ethnic minorities, and developing countries.^2,6^ While much of the research has focused on females due to the higher prevalence and sex-related aspects of eating disorders, recent studies have highlighted growing concerns about DE in males, often linked to body image issues related to muscularity and athletic performance.^7^

A wealth of literature suggests that the etiology for DE is multifactorial with individual, interpersonal, behavioral, sociocultural, biological, genetic, psychological, and environmental influences.^1^ Identifying high-risk groups for DE in adolescents is essential for early intervention and prevention.

Data from cross-sectional and some longitudinal studies suggest a role of early pubertal timing in the development of symptoms of DE.^8^ Puberty is a critical developmental phase characterized by a complex interplay of hormonal, physical, and psychological changes that transition an individual from childhood to sexual maturity and reproductive capability. This phase is marked by substantial between-person differences in the timing of pubertal onset.^8^ Worldwide, there has been a secular trend towards earlier onset of puberty in both males and females, and increased prevalence of the diagnosis of young pubertal onset.^9,10^ This is also true for Israel, where a decline in age at menarche has been documented in Jewish and Arab girls.^11,12^ Early pubertal timing often exposes adolescents to a mismatch between rapid physical development and slower emotional and cognitive growth, potentially leading to increased mental health problems.^8,13^

In a meta-analysis including predominantly females, an association between early pubertal timing and symptoms of eating disorders (e.g., body dissatisfaction and dieting) among adolescents was observed, with early pubertal timing hypothesized to lead to an increased risk of DE behaviors.^13^ However, accurately assessing pubertal stages is challenging, which complicates quick and straightforward evaluations. Therefore, proxy measures like age at menarche in girls and the appearance of facial hair in boys offer practical alternatives for gauging early pubertal maturation (EPM).^14,15^ It is important to note that menarche, which occurs later in female pubertal development, represents only one aspect of a broader developmental process.^16^ Similarly, while facial hair development in boys is associated with the later stages of puberty, it is not part of the formal stages of pubertal progression, as facial hair begins to grow approximately two years after the onset of pubarche.^17^ Nevertheless, both markers can serve as useful indicators of EPM due to their ease of self-reporting.

Earlier onset of menses has been associated with body dissatisfaction and binge eating in young adult women.^8^ To the best of our knowledge, the association between facial hair development in boys and DE remains underexplored.

Despite the high prevalence of DE in Israel,^4,5^ no prior research has explored the link between earlier pubertal maturation and DE in the country. Furthermore, although the 5-item Sick, Control, One, Fat, Food (SCOFF) questionnaire is the most widely used tool for assessing DE,^6,18,19^ it has not yet been extensively applied to this question so far. To date, only a few studies have been conducted that explore the effects of pubertal timing on DE in boys, and results are inconsistent.^8^ Additionally, there is a need for more studies using large, nationally representative samples, with adequate adjustment for confounders, to provide more comprehensive insights on this issue.

This study aimed to evaluate the associations between EPM and DE symptoms in Israeli female and male adolescents, using a well-established and easy-to-administer survey, the 5-item SCOFF questionnaire, and adjusted for potential confounders.

## Methods and study population

### Study design and sample

This study analysed data from adolescents participating in the Israel Youth Health and Nutrition Survey, carried out between the years 2015–2016 by the Israel Center for Disease Control. The design and operation of this survey have been described in detail before.^20^ In short, this was a nationally, representative school-based survey, which included 7th to 12th grade students (aged 12-–18 years). The sample schools were randomly drawn from the Israeli Ministry of Education’s school list. Schools’ compliance was high: 217/234 (92.7%), and the rate of students surveyed within schools was 5235/7029 (74.5%), primarily due to nonattendance on the day of the survey. The survey questionnaire was self-administered, and the students were measured for anthropometric data by trained personnel.

**Fig. 1:**
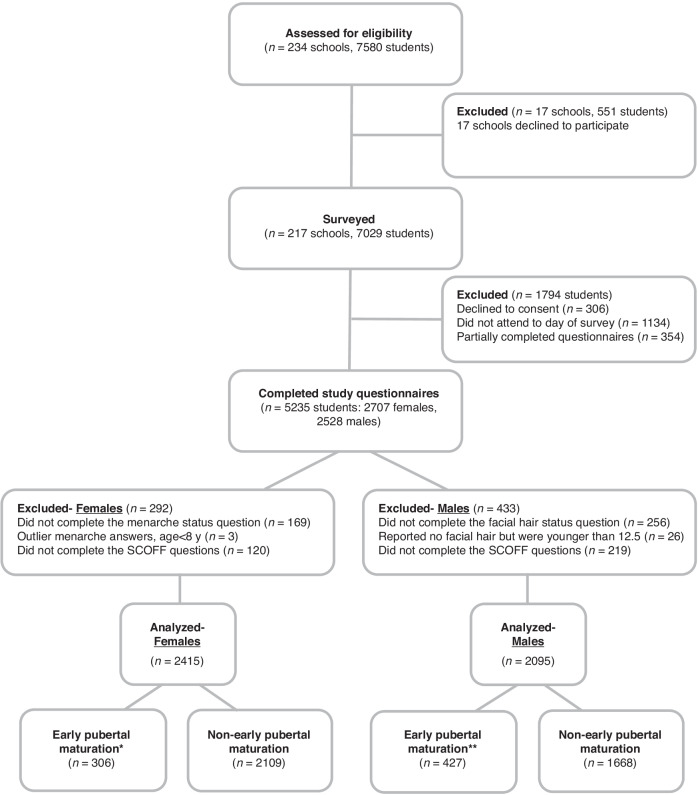
Flowchart of included study participants. *Early pubertal maturation defined as age at menarche <11.5 years, and non-early as ≥11.5. **Early pubertal maturation was defined as age at facial hair appearance <12.5 years, and non-early as ≥12.5.

The current analyses included 4510 participants (2415 girls and 2095 boys) who responded to specific questions regarding their pubertal maturation (age of menarche in girls or facial hair appearance in boys), as well as DE, according to SCOFF. Of the 5235 survey participants, 292 girls (10.8%) and 433 (17.1%) boys had missing data, and therefore were excluded from analyses (Fig. 1).

Compared to those who were not included in the analyses, those who were included were slightly older, were more likely to be girls, Jews, and from high socioeconomic status (SES) (Supplementary Table 1).

### Demographics and weight status

Age and sex were categorized by students’ responses. The Israeli Ministry of Education’s language classification of the school (Hebrew/Arabic) was used to define the population group (Jews/Arabs), as cultural, dietary, and social differences between these groups may influence health behaviors and outcomes, including DE patterns.^21^ SES was assessed using the Welfare Index rank of financial support required by the school, as identified by the Israel Ministry of Education. This index is based on family income per capita (20%), the education level of the most educated parent (40%), school location (20%), and a combination of immigration status and origin from a distressed country (20%). The welfare level is computed at the individual student level, and the Welfare Index for a specific school is the average of the scores of the registered students. The welfare level is ranked from 1 (highest) to 10 (lowest). Low SES was defined as any school ranked from 7 to 10. Schools ranked from 1 to 6 were defined as medium-high SES, as previously reported.^12^

Participating students were measured for weight using a calibrated digital scale and for height using a stadiometer, both administered by trained research personnel. BMI z-scores were calculated, and category of weight status was determined based on the World Health Organization growth standards (WHO): “underweight” < −2 standard deviations (SD), “normal weight” ≥ ( − 2) to 1 SD, “overweight” ≥1 to 2 SD, and “obese” ≥2 SD.^22^ Weight status was used as a categorical variable (overweight/obese vs. normal weight) rather than as a continuous measure such as BMI z-scores. This approach was based on the assumption that small, continuous changes in BMI are likely to have less impact compared to the broader influence of weight status categories, especially since the significance of a one-unit change in BMI can vary across different BMI ranges. Weight status categories, therefore, may better capture relevant health risks.

### DE assessment

DE symptoms were assessed using the SCOFF questionnaire,^18^ a validated screening tool for eating disorder risk and for DE among adolescents and young adults of both sexes.^6,19,23^

The SCOFF questionnaire, developed by Morgan et al. in 1999, is the most widely used screening tool for detecting eating disorders.^6,18^ This questionnaire is comprised of five yes/no questions: (1) Do you make yourself Sick because you feel uncomfortably full? (self-induced vomiting); (2) Do you worry you have lost Control over how much you eat? (loss of control over eating); (3) Have you ever lost more than One stone ( = 6.35 kg) in weight over a three-month period? (weight loss); (4) Do you believe yourself to be Fat when others say you are too thin? (body image distortion); (5) Would you say that Food dominates your life? (high impact of food on life). A positive result is indicated when the participant responds affirmatively to 2 or more questions, suggesting the presence of an eating disorder (i.e., DE).^18^

Previous systematic reviews have evaluated the SCOFF questionnaire as a screening tool within primary care settings. A systematic review and meta-analysis of 25 validation studies found the questionnaire’s cutoff of 2 or more affirmative answers to demonstrate strong validity across samples, with a pooled sensitivity of 86% and specificity of 83%.^19^ Among adolescents, prior studies have reported sensitivity ranging from 64.1 to 81.9% and specificity ranging from 77.7 to 87.2% using the same cutoff.^24–26^ Compared to more comprehensive measures such as the Eating Disorder Examination Questionnaire (EDE-Q) and the Eating Attitudes Test (EAT-26), the SCOFF is noted for its brevity and ease of use, making it a more practical option for quick screening in primary care settings.^27^ While the SCOFF is less detailed than these measures, it has been shown to effectively differentiate between adolescents with and without eating disorders in validation studies, further establishing its utility as a preliminary screening tool for identifying DE behaviors.^28–30^

In the Israel Youth Health and Nutrition Survey, SCOFF Question # 3 was modified to weight loss of 3 kg rather than one stone accommodating for the generally lower body mass of adolescents compared to adults.^20^ As suggested by Morgan et al., a total score of two or more positive answers was considered to be a case of DE.^18^ In addition, each of the five SCOFF items were analysed separately.

In order to support the accuracy of the SCOFF assessment in this study, correlations between DE (based on SCOFF score) and weight dissatisfaction (r = 0.33) and dieting intentions (r = 0.45) were evaluated using Spearman correlation (*p* < 0.001 for both). These correlations remained when analyzed by sex.

### Pubertal maturation status

To define pubertal maturation status, girls were asked: “Have you started to menstruate?”. Postmenarcheal girls were then asked, “At what age did you start to menstruate?” to identify their age at the time of their first period. EPM was determined by menarcheal age <11.5 years, in line with previous classifications.^31,32^ Girls who had not yet begun menstruating at the time of the survey were included in the non-EPM group as the study included girls aged 12 and older, with EPM defined as menarche occurring before the age of 11.5 years. Boys were asked: “At what age did facial hair first appear (e.g., moustache or beard)?” In this study, the appearance of facial hair before the age of 12.5 years was classified as early EPM, which is one year later than the age defined for EPM in girls, as previously reported.^17^ Regarding boys who had not yet developed facial hair at the time of the survey, those aged 12.5 years and above were included in the non-EPM group. However, boys younger than 12.5 years without facial hair could not be classified into either group and were therefore excluded from the study (Fig. 1).

### Statistical analyses

To represent the study population, descriptive analyses were performed and calculated with the application of the survey weights (sex, population group, and SES). Variables were presented as means and SD or as frequencies and percentages. Differences between study groups (EPM vs. Non-EPM) were tested with Student’s t-test for continuous variables, and chi-square test for categorical variables. Multivariable logistic regression analyses were performed to examine the association between EPM and DE, and between EPM and each of the SCOFF items separately. These multivariable regressions included demographic variables (Model A): age (years), SES (low versus high), population group (Jews versus Arabs). Model B included demographics and weight status (overweight/obesity versus normal weight). Since only a few (0 girls, 7 boys) in the EPM groups were defined as underweight, this category was not included in the statistical regressions.

Sensitivity analyses were conducted, testing the associations of alternative definitions of EPM with DE for each individual SCOFF item and DE by SCOFF score. The first sensitivity analysis included girls with age at menarche <12.0 years versus those with age at menarche ≥12.0 years, and boys with age at facial hair appearance <13.0 years versus those with age at facial hair appearance ≥13.0 years. Boys who had not yet developed facial hair by the time of the survey and were aged 13 years or older were categorized in the non-EPM group. Those under 13 years without facial hair could not be classified into either group and were therefore excluded from this specific analysis.

The second sensitivity analysis used three categories: < −1 SD from the mean age of menarche in girls or facial hair development in boys (earlier pubertal maturation group), −1 ≤ SD < 1 (normal pubertal maturation group, serving as the reference group), and ≥ 1 SD (later pubertal maturation group).^32^ Girls or boys who had not yet reached menarche or developed facial hair at the time of the survey were classified into the later pubertal maturation group if their age exceeded the late cutoff point; otherwise, they were excluded from this specific analysis due to the inability to determine their pubertal status.

Analyses were performed using the SAS® 9.4 program (SAS Institute Inc. 2013. Cary, NC). All statistical tests were 2-tailed and *p* < 0.05 was considered statistically significant.

### Ethical approval

The Israel Youth Health and Nutrition Survey was reviewed and approved by Sheba Medical Center’s Ethics Committee. According to the Ministry of Education guidelines, parents received written information about the survey. Children were not included if their parents objected to their offspring’s participation and consequently signed a standard form. All students had the right to refuse to participate in any part of the survey.^20^

## Results

In total, 4510 adolescents with mean age 15.2 ± 1.6 years (51.1% girls) were included in the study. They were predominantly Jews (77.5%) with 37.6% from low SES. The majority had normal weight; about 31% of both girls and boys were classified as overweight/obese (BMI ≥ 1 SD). Among the study participants, 12.7% of girls, and 20.4% of the boys met the study criteria for EPM.

DE was found in 44.9% of the youth; 55.5% in females and 33.7% in males. The rates of positive answers to each one of the 5 items in the SCOFF questions ranged from 11.3% to 50.7% in girls, and from 9.4% to 37.5% in boys (Table 1).

**Table 1:** Demographics, anthropometrics, and disordered eating symptoms of the study participants.

Table 1a: Girls^a^					
Variable	N total	All (N = 2415)	Early menarche (N = 306)	Non-early menarche (N = 2109)	*p*-value
***Demographics***					
Age, years	2415	15.2 ± 1.6	15.0 ± 1.6	15.2 ± 1.6	**0.037**
Socioeconomic status, % low	2415	980 (37.9)	125 (39.5)	855 (37.7)	0.55
Population group, % Jews	2415	1536 (76.8)	201 (78.9)	1335 (76.5)	0.35
***Anthropometrics and menarche***					
Age at menarche, years	2108	12.5 ± 1.1	10.7 ± 0.5	12.7 ± 0.9	**<0.001**
Body mass index, z-score	2168	0.44 + 1.08	0.83 ± 1.04	0.36 ± 1.07	**<0.001**
Weight status:	2168				**<0.001**
Underweight, %		29 (1.5)	0 (0)	29 (1.7)	
Normal weight, %		1485 (69.0)	159 (59.0)	1326 (70.4)	
Overweight, %		494 (22.6)	73 (27.4)	421 (21.9)	
Obese, %		160 (7.0)	36 (13.6)	124 (6.1)	
***Disordered eating by individual SCOFF items and total score***					
Do you make yourself sick because you feel uncomfortably full? %	2210	244 (11.3)	52 (18.9)	192 (10.2)	**<0.001**
Do you worry you have lost control over how much you eat? %	2404	1206 (50.7)	168 (55.6)	1038 (50.0)	0.07
Have you ever lost more than 3 kg in weight over a three-month period? %	2404	1027 (42.1)	156 (51.4)	871 (40.7)	**<0.001**
Do you believe yourself to be fat when others say you are too thin? %	2397	932 (38.3)	136 (43.9)	796 (37.5)	**0.036**
Would you say that food dominates your life? %	2403	895 (37.3)	123 (41.2)	772 (36.8)	0.14
Disordered eating ( ≥ two positive responses), %	2415	1348 (55.5)	197 (64.7)	1151 (54.2)	**0.001**

Table 1b: Boys^b^					
Variable	N total	All (N = 2095)	Early facial hair appearance (N = 427)	Non-early facial hair appearance (N = 1668)	*p*-value
***Demographics***					
Age, years	2095	15.3 ± 1.7	14.4 ± 1.6	15.5 ± 1.6	**<0.001**
Socioeconomic status, % low	2095	802 (37.1)	142 (33.1)	660 (38.1)	0.06
Population group, % Jews	2095	1514 (78.3)	279 (69.8)	1235 (80.3)	**<0.001**
***Physical characteristics***					
Age at facial hair appearance, years	1614	13.3 ± 1.6	11.4 ± 1.0	13.9 ± 1.1	**<0.001**
Body mass index z-score	1876	0.44 + 1.17	0.55 + 1.18	0.41 + 1.16	**0.031**
Weight status:	1876				0.27
Underweight, %		39 (2.1)	7 (1.7)	32 (2.2)	
Normal weight, %		1236 (66.6)	247 (63.1)	989 (67.5)	
Overweight, %		403 (21.4)	92 (23.3)	311 (20.9)	
Obese, %		198 (9.9)	50 (11.9)	148 (9.4)	
***Disordered eating by positive individual SCOFF items and total score***					
Do you make yourself sick because you feel uncomfortably full? %	1900	186 (9.4)	48 (12.6)	138 (8.6)	**0.016**
Do you worry you have lost control over how much you eat? %	2083	575 (27.6)	125 (29.0)	450 (27.2)	0.45
Have you ever lost more than 3 kg in weight over a three-month period? %	2088	786 (37.5)	189 (43.8)	597 (36.0)	**0.003**
Do you believe yourself to be fat when others say you are too thin? %	2083	347 (16.0)	80 (17.8)	267 (15.6)	0.26
Would you say that food dominates your life? %	2092	503 (24.5)	105 (24.6)	398 (24.5)	0.95
Disordered eating ( ≥ two positive responses), %	2095	718 (33.7)	160 (36.9)	558 (33.0)	0.12

Calculated with the application of sample weights of the Israeli Youth Health and Nutrition Survey.

Early menarche defined as age at menarche <11.5 years, and non-early as ≥11.5.

In boys, early facial hair appearance was defined as age at facial hair appearance <12.5 years, and non-early as ≥12.5.

Categorical variables are expressed as n (%), and continuous variables are expressed as mean ± SD.

Bold *P* values indicate significant differences between groups (*p* < 0.05).

aDemographics, anthropometrics, and disordered eating symptoms among girls with and without early age at menarche.

^b^Demographics, anthropometrics, and disordered eating symptoms among boys with and without early facial hair appearance.

### Girls

The descriptive characteristics for the girls (*n* = 2415) according to pubertal maturation status are shown in Table 1a. The mean age of the early and comparison groups was similar (15 years), with no significant differences between the groups in SES or population distribution (Arab vs. Jewish). Mean age at menarche was about 2 years younger in the early menarcheal girls versus the comparison group (*p* < 0.001). In addition, mean BMI z-score was significantly higher in the early menarche group, with 41.0% versus 28.4% classified as overweight/obesity, respectively (*p* < 0.001).

#### The association between early menarche and DE

The frequency of DE cases was approximately 10% higher in the group of girls with EPM versus the comparison group (*p* = 0.001). Significantly higher rates were also observed in three out of the 5- SCOFF items (Table 1a). In multivariable logistic regression analysis, adjusted for age, SES and group population (Model A), early menarche was associated with 56% increased odds of DE (*p* < 0.001). Early menarche was also found to be significantly associated with 100%, 29%, 56%, and 33% increased odds of 4 individual SCOFF items: self-induced vomiting, loss of control over eating, weight loss, and body image distortion, respectively. Even when weight status was taken into account (Model B), the association between early menarche and DE remained significant, in 3 out of 5 SCOFF questions (Table 2a). Concurring results were found in the first sensitivity analysis (*n* = 2415, of which 364 were classified as EPM), using an altered cutoff point for defining early age at menarche (Supplementary Tables 2a). In the second sensitivity analysis, cutoffs were defined as <11.4 years for earlier pubertal maturation ( < −1 SD, *n* = 306) and ≥13.6 years for later pubertal maturation in girls ( ≥ 1 SD, *n* = 374), with the normal pubertal maturation group classified between 11.4 and less than 13.6 years (*n* = 1499). A similar association between earlier pubertal maturation and DE, as observed in the primary analysis, was found. No significant association was found between later pubertal maturation and DE (Supplementary Table 3a).

**Table 2:** Multivariable logistic regression analyses for the associations between early pubertal maturation and disordered eating.

Table 2a: Girls^a^				
	Model A		Model B	
Outcome: Positive SCOFF items and score	Odds ratio (95% CI)	*p*-value	Odds ratio (95% CI)	*p*-value
Do you make yourself sick because you feel uncomfortably full?	1.998 (1.426, 2.798)	**<0.001**	2.012 (1.401, 2.889)	**<0.001**
Do you worry you have lost control over how much you eat?	1.294 (1.015, 1.650)	**0.038**	1.146 (0.881, 1.490)	0.31
Have you ever lost more than 3 kg in weight over a three-month period?	1.556 (1.219, 1.986)	**<0.001**	1.484 (1.136, 1.937)	**0.004**
Do you believe yourself to be fat when others say you are too thin?	1.326 (1.040, 1.692)	**0.023**	1.313 (1.011, 1.704)	**0.041**
Would you say that food dominates your life?	1.197 (0.935, 1.532)	0.15	1.200 (0.921, 1.563)	0.18
Disordered eating ( ≥ two affirmative responses)	1.564 (1.216, 2.010)	**<0.001**	1.466 (1.116, 1.925)	**0.006**

Table 2b: Boys^b^				
Do you make yourself sick because you feel uncomfortably full?	1.278 (0.889, 1.837)	0.19	1.207 (0.814, 1.789)	0.35
Do you worry you have lost control over how much you eat?	1.099 (0.861, 1.404)	0.45	0.973 (0.745, 1.272)	0.84
Have you ever lost more than 3 kg in weight over a three-month period?	1.509 (1.204, 1.889)	**<0.001**	1.477 (1.147, 1.902)	**0.003**
Do you believe yourself to be fat when others say you are too thin?	1.074 (0.805, 1.434)	0.63	0.942 (0.688, 1.289)	0.71
Would you say that food dominates your life?	1.171 (0.904, 1.517)	0.23	1.197 (0.909, 1.575)	0.20
Disordered eating ( ≥ two affirmative responses)	1.127 (0.895, 1.419)	0.31	0.996 (0.773, 1.284)	0.98

Results-Odds ratios (95% CI) for early vs. non-early pubertal maturation signs in girls defined as age at menarche <11.5 years versus age at menarche ≥11.5 as reference (odds ratio = 1); in boys: age at facial hair appearance <12.5 vs. ≥12.5 as reference (odds ratio = 1).

Model A: adjusted for age, socioeconomic status, and population group.

Model B: adjusted for age, socioeconomic status, population group, and weight status.

Bold *P* values indicate significant associations (*p* < 0.05).

*DE* disordered eating, *CI* confidence interval.

^a^Multivariable logistic regression analyses for the associations between early age at menarche among girls and DE for each individual SCOFF items (separately) and DE by SCOFF score.

^b^Multivariable logistic regression analyses for the associations between early facial hair appearance among boys and DE for each individual SCOFF items (separately) and DE by SCOFF score.

### Boys

Table 1b presents the descriptive characteristics for males (*n* = 2095), according to pubertal maturation status. Those classified with EPM were more likely to be younger, Arab and of high socioeconomic status (*p* < 0.05, for all). Mean age of facial hair appearance was about 2.5 years younger in EPM group versus the comparison group (*p* < 0.001). In the boys with EPM mean BMI z-score was significantly higher. The prevalence of overweight/obesity tended to be higher in those with EPM at 35.8% versus 31.0% among their peers (*p* = 0.07).

#### The association between early facial hair appearance and DE

No significant differences were found in the prevalence of DE cases, nor in three out of the 5 SCOFF items between the boys with or without early facial hair appearance (all *p* values > 0.05). The two questions with a significantly higher rate of affirmative answers in boys with EPM were the self-induced vomiting (*p* = 0.016) and the weight loss (*p* = 0.003) questions. Controlling for demographics (Model A), and also for weight status (Model B), EPM in boys was associated with 50% and 48% increased odds for the weight loss SCOFF item, respectively, but not with DE or other SCOFF questions (Table 2b). The first sensitivity analysis (*n* = 1981, of which 499 were classified as EPM) indicated that results were unchanged when using an altered cutoff points for defining early facial hair appearance (Supplementary Table 2b). In the second sensitivity analysis, cutoffs were defined as <11.7 years for earlier pubertal maturation ( < −1 SD, *n* = 168) and ≥14.9 years for later pubertal maturation in boys ( ≥1 SD, *n* = 411), with the normal pubertal maturation group classified between 11.7 and less than 14.9 years (*n* = 1156). No significant association was found between earlier pubertal maturation and DE, similar to the primary analysis. Additionally, no significant association was found between later pubertal maturation and DE (Supplementary Table 3b).

## Discussion

In a large sample of Israeli youth, DE, defined as two or more positive answers on the SCOFF questionnaire, was widespread (44.9%) in the population. Rates in females were higher than in males, 55.5% and 33.7% respectively. In females, EPM, defined by early menarcheal age, was associated with DE. This result was in line with the study hypothesis. After controlling for potential confounders, early menarcheal age was also significantly associated with 3 out of 5 SCOFF items; body image distortion, self-induced vomiting, and weight loss. Additionally, an association between EPM, as defined by age of appearance of facial hair, and weight loss was identified among Israeli boys. However, since this is a cross-sectional study, the direction of the associations found cannot be definitively determined.

The prevalence of DE is known to be high in Israel.^4,5,33^ Results from a recent, large (*n* = 63,181) international, meta-analysis of DE in 16 countries, using the SCOFF questionnaire, reported DE rates of 30.0% (95% CI: 25.6%–34.7%) in girls and 17.0% (95% CI: 13.5%–20.8%) in boys.^6^ A few countries reported similar data to those found in Israel. A high percentage of DE was observed in Saudi Arabia, Singapore and Slovenia. In these countries, rates of DE in girls ranged from 45.6%-54.2% and for boys rates ranged from 24.8%–47.2%. However, it should be noted, that in the current study, it is possible that the rates of DE were overestimated. Modification of Question #3 in the Israeli SCOFF questionnaire from weight loss of 1 stone to 3 kg could possibly lead to a disparity in prevalence rates.

The association between EPM and DE has been documented in the literature, primarily in girls.^8,34^ A meta-analysis by Ullsperger and Nikolas (2017) found that early pubertal timing increased the risk of eating pathologies, however, definitions for both variables differed among studies.^13^ Results reported here are in line with these results and show that EPM in Israeli girls was associated with a 1.47-fold increased odds (95% CI: 1.12–1.93) of DE. Sensitivity tests further confirmed this conclusion. This result also aligns with previous data from the Israel Youth, Health and Nutrition Survey carried out in 2003–4.^4^ In the earlier survey, it was observed that in girls with age at menarche of 12 years or less there was a 1.43-fold higher odds (95% CI: 1.17–1.75) of DE symptoms.

The observation that EPM was significantly associated with certain SCOFF items but not others may stem from the varied ways in which specific DE behaviors are linked to pubertal timing. This observation is similar to the findings by Shope et al. ^8^, where early pubertal timing was associated with specific symptoms such as body dissatisfaction and binge eating but not others (e.g., dieting, excessive exercise). In girls, significant associations between early menarche and behaviors like self-induced vomiting, weight loss, and body image distortion were found. These behaviors are closely tied to body dissatisfaction, which is often heightened during early physical maturation, particularly in the context of societal pressures to conform to thinness ideals.^35^ The two SCOFF questions that did not show a significant association with early menarche were those related to the fear of losing control over food intake and the feeling that food dominates one’s life. Both questions revolve around the theme of control, a critical component in certain eating disorders. These control-related behaviors and perceptions may be multifaceted and influenced by a variety of factors beyond the physical changes of puberty, thereby increasing their complexity.

In contrast, among boys, EPM (appearance of facial hair before age 12.5 years) was positively associated with a single SCOFF item (weight loss). Analysis of the entire SCOFF score did not yield significant results. These findings were confirmed in sensitivity analyses. Overall, previous findings in boys have been inconsistent, showing mixed results regarding the association between pubertal maturation timing and DE.^34^ Some DE symptoms, such as weight/shape concerns, appear to be positively associated with EPM.^34^ In this study, the significant association found between EPM and weight loss may stem from efforts to reduce fat mass in pursuit of a more muscular body, a concern that tends to increase during early physical development in males. However, with regard to other DE behaviors and perceptions, such as self-induced vomiting, the association with EPM may be weaker. This could be because these behaviors may not be as directly influenced by the changes of puberty, and might instead be more affected by psychological, social, or cultural factors that could accompany developmental transitions. It is of interest to note that research supports associations between muscularity-oriented body image concerns and DE in males.^7,36,37^ However, the SCOFF questionnaire does not specifically address this issue.

Several conceptual models have been hypothesized to explain the relationship between EPM and psychopathology. The hypothesis that has received the most empirical support is the “maturation disparity hypothesis”, which has also been termed the “developmental readiness hypothesis”.^38^ This hypothesis assumes that early adolescents experience psychological distress due to a mismatch between their fast physical development and slower progressive emotional and cognitive development, which may lead to increased mental health problems.^13^ In light of this conceptual model it was hypothesized that youth who mature early, experience physical changes before their peers and therefore may experience additional body dissatisfaction then their counterparts. Body dissatisfaction is associated with attempts at weight loss.^34^ Girls are considered more prone to DE relative to boys, possibly because pubertal changes in girls are related to increased adiposity and move them away from the female thin ideal. In contrast, physical changes in boys during puberty often involve increased muscle mass which actually moves them closer to their ideal body appearance.^34^

It has been found in previous studies that early menarche was associated with high BMI and with higher levels of DE.^8^ These findings align with the “maturation disparity hypothesis”, which posits that a higher BMI is particularly risky for those experiencing early puberty, as their cognitive-emotional system is less developed during this period, hindering adaptive coping.^8,13^ In the current study, the prevalence of overweight/obese girls was significantly higher in the early menarche group compared to controls (41.0% vs. 28.4%; *p* < 0.001). However, when controlling for weight status (Model B) the relationship between early puberty and DE remained significant in girls. This suggests that BMI/weight status alone cannot explain the association between early menarche and DE, indicating that other variables are involved.

Hormones secreted during puberty are thought to play a critical role in sex-differentiated behaviors. Therefore, they are the focus of much research attempting to identify hormonal factors underlying pubertal association with DE. Additionally, genetic factors may also play a role. A longitudinal study of 924 sister pairs demonstrated that common genetic influences accounted for the association between early menarcheal age and an increased risk for dieting during adolescence.^39^ Research on twins suggests that genetic factors influencing a younger age at menarche are associated with a greater susceptibility to DE.^34,40^ Shared genetic mechanisms likely involve ovarian hormones, particularly estradiol, which regulate both pubertal timing and behaviors associated with DE, such as body dissatisfaction and dietary restraint. Furthermore, genetic variations in serotonergic and dopaminergic pathways—which influence emotional regulation, mood, and reward sensitivity—are also implicated.^41^

While the current study hypothesizes that early menarche may lead to DE, it is also important to consider the potential for a reverse association between DE and EPM. DE behaviors, particularly those that result in increased BMI, could themselves influence the timing of pubertal onset. Studies have shown that higher BMI, often associated with DE patterns such as binge eating, can accelerate pubertal development, including early menarche.^42^ This association could be mediated by increased levels of leptin, a hormone involved in both energy regulation and the onset of puberty, which tends to be elevated in individuals with higher body fat.^43,44^ Future longitudinal research is necessary to clarify the directionality of this relationship.

The primary aim of this study was to explore the specific association between EPM and DE. However, the second sensitivity analysis also provided insight into later pubertal maturation, revealing the lack of association between later pubertal maturation and DE in both boys and girls in the current study. The results for girls are consistent with those of Yannakoulia et al., who demonstrated a weak negative association between the age of menarche and the total EAT score.^45^ It is important to note that DE itself could contribute to delayed menarche, particularly through mechanisms such as energy deficiency and low body fat, which are crucial for normal pubertal development.^46^ In this study, we did not observe such an association, which may be due to the fact that the age threshold defined as “later” was not sufficiently advanced.

A systematic review by Byrne et al. noted that among boys, increased concerns about muscularity, weight, and body shape, along with overweight or underweight status, were significant predictors of eating attitudes, dietary restraint, and muscularity-oriented behaviors over time.^47^ These concerns may be further exacerbated in cases of late pubertal development. However, the current study did not find an association between later pubertal development and DE in boys. This lack of association may be attributed to the relatively low age cutoff for later pubertal maturation used in the study or to cultural factors that influence adolescents’ perceptions of body image, shaping the manifestation of DE behaviors.

It is worth noting that higher cutoffs for later pubertal maturation in both boys and girls resulted in a limited number of participants with significantly later maturation, making it challenging to establish firm conclusions. For instance, only 18 girls (0.7%) and 68 boys (3.2%) reported menarche/facial hair appearance after the ages of 15.5 and 16.5 years, respectively.

The findings from the second sensitivity analysis were consistent with those of the primary analysis regarding earlier maturation, showing a similar association with DE when compared to the normal group alone, rather than to the rest of the cohort.^32^ Additionally, the second sensitivity analysis method allowed for a relative classification of pubertal maturation based on the sample’s data, providing a more precise reflection of the study population.

As the prevalence of DE has been increasing over the years, the importance of screening adolescents in the general population for DE behaviors and perceptions becomes more critical.^6^ Identifying risk groups for DE and eating disorders is essential and should be implemented effectively taking into account limited resources. Establishing the association between early menarche and DE in Israeli adolescents indicates that a single, simple question can identify a group with a significantly increased prevalence of disordered behaviors/attitudes, without the need to delve into the detailed stages of puberty. Early detection of DE enables tailored treatment and guidance for the adolescent, their family, and their environment. Thus, preventing the condition from worsening and improving overall well-being. However, in boys, no relationship was found between early age of the appearance of facial hair and DE. This highlights the need for continued research to develop straightforward screening questions to effectively identify at-risk males.

### Limitations and strengths

This study had some limitations. Causal relationships could not be established due to the cross-sectional design. Selection bias may also be a limitation as some characteristics of the study sample differed from those of participants who were not included in the analyses. Furthermore, the study relied largely on self-report, which can bias answers. However, it has been shown that self-reports of age at menarche are reliable and valid.^48^ Additionally, use of the SCOFF questionnaire may underestimate DE in males. Lastly, the use of a single indicator to measure pubertal timing for both males and females—age at menarche for girls and facial hair development for boys. These measures capture only specific aspects of the overall pubertal process. However, it is also important to recognize the value of simple and feasible tools, particularly in community-based settings, where quick screening methods are essential. Future studies would benefit from employing multiple indicators to provide a more nuanced understanding of pubertal timing and its relationship with DE behaviors.

Study strengths include a large sample of both male and female adolescents, participating in the Israeli National Youth Health and Nutrition Survey. Extensive demographic and other data were available, which allowed for statistical control in analyses. In addition, weight and height measurements were carried out by trained personnel, providing reliable data for these variables. Finally, DE was evaluated by the SCOFF questionnaire, a well-established and long-standing tool that has been used in several adolescent population-based surveys.^6^ Sensitivity analyses were carried out to confirm the reported associations.

## Conclusion

The prevalence of DE among Israeli adolescents is concerning since more than half of the girls and a third of the boys were characterized as having DE symptoms. Consistent with several other studies, this study also indicated that EPM presents a significant correlate for DE in adolescent girls. Prevention strategies such as screening for DE by health professionals (e.g., pediatricians, endocrinologists, dietitians) are recommended, especially for girls with early menarche, who should be considered a high-risk group for DE. Further longitudinal prospective studies are needed to establish the causal relationships between early EPM and DE in adolescent girls and boys, and examine possible mediators in these relationships.

## Supplementary information

Supplemental Table 1

Supplemental Table 2

Supplemental Table 3

## Data Availability

Data will be provided upon request and in accordance with the decision of the Israel Center for Disease Control’s Publications Committee.
