# Supplementary material for: Early menarche is associated with disordered eating—results from a National Youth Survey
Source: Pediatr Res. 2025 Jan 16;98(3):909–17. doi: 10.1038/s41390-025-03852-1 (PMC12507651; doi:10.1038/s41390-025-03852-1)
Supplement: Supplementary file 1 — Supplemental Table 1 [file 41390_2025_3852_MOESM1_ESM.pdf]

**Supplemental Table 1.** Characteristics of participants included compared to those not included in the analyses

| **Variable** | **Participants included in the analyses (N=4510)** | **Participants not included in the analyses (N=725)** | **p-value** |
|---|---|---|---|
| Age, years | 15.2 ± 1.6 | 14.9 ± 1.8 | <.001 |
| Sex, % males | 2095 (48.9) | 433 (63.2) | <.001 |
| Socioeconomic status, % low | 1782 (37.6) | 354 (47.1) | <.001 |
| Population group, % Jews | 3050 (77.5) | 347 (58.4) | <.001 |
| Body mass index z-score | 0.43 ± 1.12 | 0.59 ± 1.23 | 0.001 |
| Weight status, % overweight/obesity | 1255 (31.0) | 226 (37.4) | 0.003 |

Note: Calculated with the application of sample weights of the Israeli Youth Health and Nutrition Survey.

Categorical variables are expressed as n (%), and continuous variables are expressed as mean ± SD.
